# Abacavir methanol 2.5-solvate

**DOI:** 10.1107/S1600536809027743

**Published:** 2009-07-22

**Authors:** Phuong-Truc T. Pham

**Affiliations:** a Department of Chemistry, Penn State Worthington Scranton, 120 Ridge View Drive, Dunmore, Pennsylvania 18512, USA

## Abstract

The structure of abacavir (systematic name: {(1*S*,4*R*)-4-[2-amino-6-(cyclo­propyl­amino)-9*H*-purin-9-yl]cyclo­pent-2-en-1-yl}methanol), C_14_H_18_N_6_O·2.5CH_3_OH, consists of hydrogen-bonded ribbons which are further held together by additional hydrogen bonds involving the hydroxyl group and two N atoms on an adjacent purine. The asymmetric unit also contains 2.5 mol­ecules of methanol solvate which were grossly disordered and were excluded using SQUEEZE subroutine in *PLATON* [Spek, (2009 ▶). *Acta Cryst*. D**65**, 148–155].

## Related literature

For a related structure, see: Huang *et al.* (2007 ▶). For the synthesis, see: Vince & Hua (1990 ▶). For an X-ray powder diffraction analysis of abacavir hemisulfate, see: Monger & Varlashkin (2005 ▶).
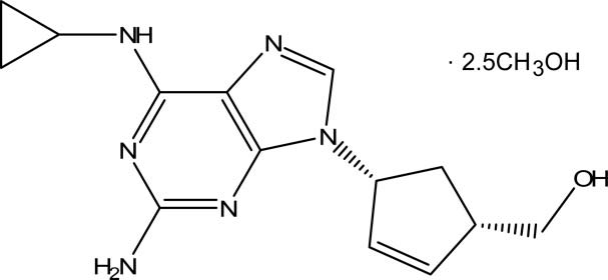


## Experimental

### 

#### Crystal data

C_14_H_18_N_6_O·2.5CH_4_O
*M* *_r_* = 366.45
Monoclinic, 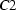

*a* = 19.857 (4) Å
*b* = 7.2552 (15) Å
*c* = 13.735 (3) Å
β = 98.27 (3)°
*V* = 1958.2 (7) Å^3^
*Z* = 4
Mo *K*α radiation
μ = 0.09 mm^−1^
*T* = 173 K
0.60 × 0.30 × 0.15 mm

#### Data collection

Bruker SMART Platform CCD diffractometer
Absorption correction: multi-scan (*SADABS*; Bruker, 2003 ▶) *T*_min_ = 0.948, *T*_max_ = 0.987
10335 measured reflections
2405 independent reflections
2231 reflections with *I* > 2σ(*I*)
*R*_int_ = 0.024

#### Refinement

*R*[*F* ^2^ > 2σ(*F* ^2^)] = 0.036
*wR*(*F* ^2^) = 0.099
*S* = 1.01
2405 reflections
190 parameters
1 restraint
H-atom parameters constrained
Δρ_max_ = 0.18 e Å^−3^
Δρ_min_ = −0.19 e Å^−3^

### 

Data collection: *SMART* (Bruker, 2003 ▶); cell refinement: *SAINT* (Bruker, 2006 ▶); data reduction: *SAINT*; program(s) used to solve structure: *SHELXS97* (Sheldrick, 2008 ▶); program(s) used to refine structure: *SHELXL97* (Sheldrick, 2008 ▶); molecular graphics: *SHELXTL* (Sheldrick, 2008 ▶); software used to prepare material for publication: *SHELXTL*.

## Supplementary Material

Crystal structure: contains datablocks I, global. DOI: 10.1107/S1600536809027743/pv2180sup1.cif

Structure factors: contains datablocks I. DOI: 10.1107/S1600536809027743/pv2180Isup2.hkl

Additional supplementary materials:  crystallographic information; 3D view; checkCIF report

## Figures and Tables

**Table 1:** Hydrogen-bond geometry (Å, °)

*D*—H⋯*A*	*D*—H	H⋯*A*	*D*⋯*A*	*D*—H⋯*A*
N1—H1*A*⋯N4^i^	0.88	2.16	3.036 (2)	177
N1—H1*B*⋯O1^ii^	0.88	2.36	3.036 (2)	134
N3—H3*N*⋯N5^iii^	0.88	2.21	3.016 (2)	152
O1—H1*O*⋯N2^iv^	0.84	2.05	2.808 (2)	150

Symmetry codes: (i) 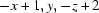; (ii) 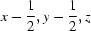; (iii) 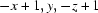; (iv) 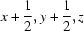.

**Fig. 1.**
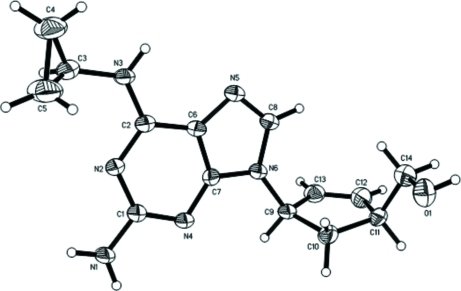
The molecular structure of abacavir with atomic lables; thermal displacement ellipsoids have been plotted at 50% probability level.

**Fig. 2.**
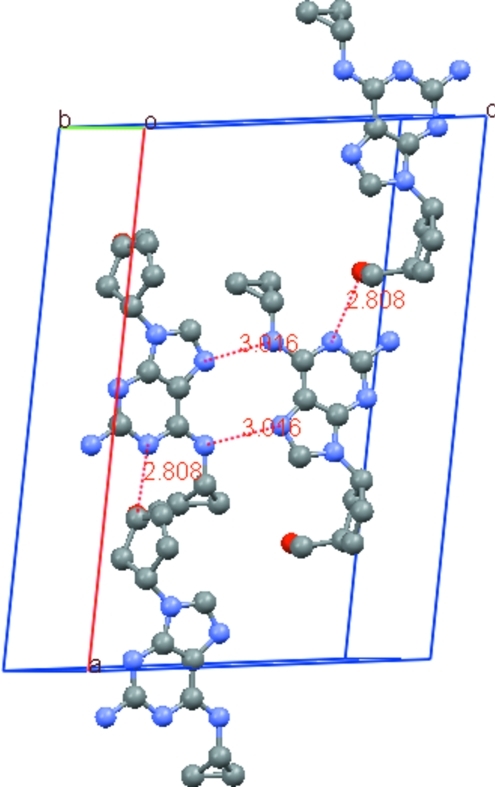
Crystal packing of abacavir molecules in the unit cell viewed along the a axis.

## supplementary crystallographic information

### Comment

Abacavir is a potent anti-HIV drug which acquires its activity through inhibiting the viral reverse transcriptase. The crystal structure of this biologically important drug is not known. An *X*-ray powder diffraction analysis of abacavir hemisulfate, however, has been reported (Monger & Varlashkin, 2005). The structure of abacavir (Fig. 1) contains wide cylindrical channels that are parallel to the *c* axis (Fig. 2). The lattice is held together by hydrogen bonds (details are in Table 1). The absolute configuration around C9 and C11 of abacavir was assigned as *R* and *S*, respectively, based on the synthetic procedures. The large voids in the lattice of abacavir appear to hold methanol solvate molecules but attempts to model the solvent were unsuccessful.

### Experimental

Abacavir was prepared according to literature procedure (Vince & Hua, 1990). The compound was dissolved in a minimal amount of hot methanol and the solution was then placed in a chamber saturated with dichloromethane at room temperature, covered and allowed to crystallize for two weeks. The resulting clear colorless rod shaped crystals were washed with cold methanol, dried then collected and a suitable crystal was selected for structural determination.

### Refinement

The program *PLATON* (Spek, 2009) indicated solvent accessible void space of 688.7 Å^3^, corresponding to 179 electrons in a unit cell, equivalent to ten molecules of methanol solvate. Since the solvent molecules were grossly disordered and could not be modeled, their contribution was excluded using the subroutine SQUEEZE. H atoms were placed in idealized positions and treated as riding atoms with distances: O—H = 0.84, N—H 0.88 and C—H in the range 0.95–1.00 Å and *U*_iso_(H) = 1.2*U*_eq_(parent atom). An absolute structure could not be determined by anoimalous dispersion effects; Friedel pairs (2405) were therefore merged.

### Crystal data

**Table:**

C_14_H_18_N_6_O·2.5CH_4_O	*F*(000) = 788
*M**_r_* = 366.45	*D*_x_ = 1.243 Mg m^−^^3^
Monoclinic, *C*2	Mo *K*α radiation, λ = 0.71073 Å
Hall symbol: C 2y	Cell parameters from 2936 reflections
*a* = 19.857 (4) Å	θ = 2.4–27.4°
*b* = 7.2552 (15) Å	µ = 0.09 mm^−^^1^
*c* = 13.735 (3) Å	*T* = 173 K
β = 98.27 (3)°	Rod, colorless
*V* = 1958.2 (7) Å^3^	0.60 × 0.30 × 0.15 mm
*Z* = 4	

### Data collection

**Table:**

Bruker SMART Platform CCD diffractometer	2405 independent reflections
Radiation source: normal-focus sealed tube	2231 reflections with *I* > 2σ(*I*)
graphite	*R*_int_ = 0.024
area detector, ω scans per φ	θ_max_ = 27.5°, θ_min_ = 1.5°
Absorption correction: multi-scan (*SADABS*; Bruker, 2003)	*h* = −25→25
*T*_min_ = 0.948, *T*_max_ = 0.987	*k* = −9→9
10335 measured reflections	*l* = −17→17

### Refinement

**Table:**

Refinement on *F*^2^	Primary atom site location: structure-invariant direct methods
Least-squares matrix: full	Secondary atom site location: difference Fourier map
*R*[*F*^2^ > 2σ(*F*^2^)] = 0.036	Hydrogen site location: inferred from neighbouring sites
*wR*(*F*^2^) = 0.099	H-atom parameters constrained
*S* = 1.01	*w* = 1/[σ^2^(*F*_o_^2^) + (0.0623*P*)^2^ + 0.4691*P*] where *P* = (*F*_o_^2^ + 2*F*_c_^2^)/3
2405 reflections	(Δ/σ)_max_ < 0.001
190 parameters	Δρ_max_ = 0.18 e Å^−^^3^
1 restraint	Δρ_min_ = −0.19 e Å^−^^3^

### Special details

**Table:**

Geometry. All esds (except the esd in the dihedral angle between two l.s. planes) are estimated using the full covariance matrix. The cell esds are taken into account individually in the estimation of esds in distances, angles and torsion angles; correlations between esds in cell parameters are only used when they are defined by crystal symmetry. An approximate (isotropic) treatment of cell esds is used for estimating esds involving l.s. planes.
Refinement. Refinement of F^2^ against ALL reflections. The weighted R-factor wR and goodness of fit S are based on F^2^, conventional R-factors R are based on F, with F set to zero for negative F^2^. The threshold expression of F^2^ > 2sigma(F^2^) is used only for calculating R-factors(gt) etc. and is not relevant to the choice of reflections for refinement. R-factors based on F^2^ are statistically about twice as large as those based on F, and R- factors based on ALL data will be even larger.

### Fractional atomic coordinates and isotropic or equivalent isotropic displacement parameters (Å^2^)

**Table:**

	*x*	*y*	*z*	*U*_iso_*/*U*_eq_	
O1	0.78278 (7)	0.9453 (2)	0.79352 (11)	0.0435 (4)	
H1O	0.8119	1.0020	0.7664	0.065*	
N1	0.41393 (7)	0.5553 (3)	0.92222 (10)	0.0353 (4)	
H1A	0.4354	0.5494	0.9828	0.042*	
H1B	0.3694	0.5669	0.9115	0.042*	
N2	0.41135 (7)	0.5588 (2)	0.75419 (9)	0.0265 (3)	
N3	0.40604 (7)	0.5727 (2)	0.58458 (9)	0.0302 (4)	
H3N	0.4273	0.5824	0.5328	0.036*	
N4	0.51700 (7)	0.5290 (2)	0.86615 (9)	0.0252 (3)	
N5	0.56198 (7)	0.5232 (3)	0.62195 (9)	0.0307 (3)	
N6	0.61422 (7)	0.4984 (2)	0.77840 (9)	0.0259 (3)	
C1	0.44957 (8)	0.5466 (3)	0.84530 (11)	0.0253 (3)	
C2	0.44332 (8)	0.5568 (3)	0.67411 (11)	0.0253 (3)	
C3	0.33305 (10)	0.5746 (4)	0.57052 (13)	0.0413 (5)	
H3	0.3100	0.4545	0.5796	0.050*	
C4	0.29783 (13)	0.7005 (5)	0.49353 (15)	0.0589 (8)	
H4A	0.2545	0.6579	0.4555	0.071*	
H4B	0.3265	0.7770	0.4560	0.071*	
C5	0.29839 (14)	0.7448 (5)	0.60113 (15)	0.0671 (9)	
H5B	0.3274	0.8480	0.6294	0.080*	
H5C	0.2554	0.7290	0.6289	0.080*	
C6	0.51484 (8)	0.5376 (3)	0.68753 (11)	0.0249 (3)	
C7	0.54633 (8)	0.5233 (2)	0.78383 (11)	0.0234 (3)	
C8	0.61989 (8)	0.5003 (3)	0.67988 (11)	0.0295 (4)	
H8	0.6621	0.4863	0.6558	0.035*	
C9	0.66820 (9)	0.4790 (3)	0.86407 (11)	0.0295 (4)	
H9	0.6478	0.4289	0.9210	0.035*	
C10	0.70507 (9)	0.6626 (3)	0.89483 (14)	0.0346 (4)	
H10A	0.7068	0.6837	0.9664	0.042*	
H10B	0.6811	0.7675	0.8590	0.042*	
C11	0.77807 (9)	0.6425 (3)	0.86777 (13)	0.0314 (4)	
H11	0.8127	0.6785	0.9248	0.038*	
C12	0.78314 (10)	0.4411 (3)	0.84697 (14)	0.0349 (4)	
H12	0.8245	0.3822	0.8380	0.042*	
C13	0.72425 (9)	0.3522 (3)	0.84218 (13)	0.0322 (4)	
H13	0.7182	0.2250	0.8269	0.039*	
C14	0.78838 (11)	0.7532 (3)	0.77666 (15)	0.0398 (5)	
H14A	0.8339	0.7260	0.7589	0.048*	
H14B	0.7539	0.7160	0.7208	0.048*	

### Atomic displacement parameters (Å^2^)

**Table:**

	*U*^11^	*U*^22^	*U*^33^	*U*^12^	*U*^13^	*U*^23^
O1	0.0336 (7)	0.0455 (9)	0.0523 (9)	−0.0024 (6)	0.0089 (6)	−0.0002 (7)
N1	0.0258 (7)	0.0635 (12)	0.0166 (6)	0.0035 (8)	0.0027 (5)	−0.0004 (8)
N2	0.0227 (6)	0.0382 (9)	0.0180 (6)	0.0001 (6)	0.0008 (5)	−0.0003 (6)
N3	0.0257 (7)	0.0492 (10)	0.0152 (6)	0.0062 (7)	0.0012 (5)	0.0002 (6)
N4	0.0262 (7)	0.0330 (8)	0.0159 (6)	0.0000 (6)	0.0018 (5)	0.0005 (6)
N5	0.0295 (7)	0.0447 (10)	0.0184 (6)	−0.0011 (7)	0.0048 (5)	0.0009 (7)
N6	0.0238 (6)	0.0370 (9)	0.0172 (6)	−0.0004 (6)	0.0034 (5)	0.0005 (6)
C1	0.0253 (7)	0.0303 (9)	0.0204 (7)	−0.0002 (7)	0.0032 (6)	−0.0003 (7)
C2	0.0280 (8)	0.0281 (9)	0.0191 (7)	−0.0002 (7)	0.0012 (6)	−0.0008 (7)
C3	0.0315 (9)	0.0679 (15)	0.0232 (8)	0.0054 (10)	−0.0006 (7)	0.0001 (9)
C4	0.0509 (13)	0.096 (2)	0.0273 (10)	0.0352 (15)	−0.0019 (9)	0.0021 (12)
C5	0.0587 (15)	0.112 (3)	0.0296 (11)	0.0447 (17)	0.0021 (10)	−0.0025 (14)
C6	0.0278 (7)	0.0304 (9)	0.0166 (7)	−0.0006 (7)	0.0036 (6)	0.0016 (7)
C7	0.0226 (7)	0.0273 (9)	0.0200 (7)	−0.0034 (7)	0.0018 (5)	0.0012 (7)
C8	0.0260 (8)	0.0441 (11)	0.0190 (7)	−0.0011 (8)	0.0051 (6)	0.0000 (8)
C9	0.0259 (8)	0.0459 (11)	0.0163 (7)	−0.0034 (8)	0.0017 (6)	−0.0001 (7)
C10	0.0254 (9)	0.0472 (12)	0.0316 (9)	−0.0024 (8)	0.0049 (7)	−0.0144 (8)
C11	0.0186 (8)	0.0504 (12)	0.0240 (8)	−0.0018 (7)	−0.0012 (6)	−0.0036 (8)
C12	0.0270 (9)	0.0460 (12)	0.0309 (9)	0.0075 (8)	0.0011 (7)	0.0055 (8)
C13	0.0329 (9)	0.0385 (10)	0.0245 (8)	0.0041 (8)	0.0015 (7)	0.0035 (8)
C14	0.0417 (11)	0.0444 (13)	0.0345 (10)	−0.0079 (9)	0.0097 (8)	−0.0045 (9)

### Geometric parameters (Å, °)

**Table:**

O1—C14	1.419 (3)	C4—C5	1.511 (3)
O1—H1O	0.8394	C4—H4A	0.9900
N1—C1	1.355 (2)	C4—H4B	0.9900
N1—H1A	0.8798	C5—H5B	0.9900
N1—H1B	0.8801	C5—H5C	0.9900
N2—C2	1.347 (2)	C6—C7	1.383 (2)
N2—C1	1.370 (2)	C8—H8	0.9500
N3—C2	1.346 (2)	C9—C13	1.507 (3)
N3—C3	1.434 (2)	C9—C10	1.550 (3)
N3—H3N	0.8804	C9—H9	1.0000
N4—C1	1.334 (2)	C10—C11	1.554 (2)
N4—C7	1.345 (2)	C10—H10A	0.9900
N5—C8	1.311 (2)	C10—H10B	0.9900
N5—C6	1.393 (2)	C11—C12	1.495 (3)
N6—C7	1.373 (2)	C11—C14	1.526 (3)
N6—C8	1.374 (2)	C11—H11	1.0000
N6—C9	1.480 (2)	C12—C13	1.329 (3)
C2—C6	1.412 (2)	C12—H12	0.9500
C3—C4	1.493 (3)	C13—H13	0.9500
C3—C5	1.503 (4)	C14—H14A	0.9900
C3—H3	1.0000	C14—H14B	0.9900
			
C14—O1—H1O	109.5	C7—C6—C2	116.10 (14)
C1—N1—H1A	120.0	N5—C6—C2	132.81 (14)
C1—N1—H1B	120.0	N4—C7—N6	126.66 (14)
H1A—N1—H1B	120.0	N4—C7—C6	127.64 (14)
C2—N2—C1	118.76 (13)	N6—C7—C6	105.69 (14)
C2—N3—C3	122.41 (14)	N5—C8—N6	114.20 (14)
C2—N3—H3N	118.8	N5—C8—H8	122.9
C3—N3—H3N	118.8	N6—C8—H8	122.9
C1—N4—C7	111.41 (13)	N6—C9—C13	111.75 (14)
C8—N5—C6	103.26 (13)	N6—C9—C10	113.26 (16)
C7—N6—C8	105.80 (13)	C13—C9—C10	104.22 (15)
C7—N6—C9	125.03 (13)	N6—C9—H9	109.2
C8—N6—C9	129.15 (14)	C13—C9—H9	109.2
N4—C1—N1	117.23 (14)	C10—C9—H9	109.2
N4—C1—N2	127.52 (14)	C9—C10—C11	105.93 (16)
N1—C1—N2	115.24 (14)	C9—C10—H10A	110.5
N3—C2—N2	118.91 (15)	C11—C10—H10A	110.5
N3—C2—C6	122.56 (14)	C9—C10—H10B	110.5
N2—C2—C6	118.54 (14)	C11—C10—H10B	110.5
N3—C3—C4	117.6 (2)	H10A—C10—H10B	108.7
N3—C3—C5	117.7 (2)	C12—C11—C14	109.71 (16)
C4—C3—C5	60.57 (16)	C12—C11—C10	103.19 (17)
N3—C3—H3	116.5	C14—C11—C10	112.74 (16)
C4—C3—H3	116.5	C12—C11—H11	110.3
C5—C3—H3	116.5	C14—C11—H11	110.3
C3—C4—C5	60.04 (16)	C10—C11—H11	110.3
C3—C4—H4A	117.8	C13—C12—C11	113.64 (19)
C5—C4—H4A	117.8	C13—C12—H12	123.2
C3—C4—H4B	117.8	C11—C12—H12	123.2
C5—C4—H4B	117.8	C12—C13—C9	111.33 (19)
H4A—C4—H4B	114.9	C12—C13—H13	124.3
C3—C5—C4	59.39 (16)	C9—C13—H13	124.3
C3—C5—H5B	117.8	O1—C14—C11	111.11 (17)
C4—C5—H5B	117.8	O1—C14—H14A	109.4
C3—C5—H5C	117.8	C11—C14—H14A	109.4
C4—C5—H5C	117.8	O1—C14—H14B	109.4
H5B—C5—H5C	115.0	C11—C14—H14B	109.4
C7—C6—N5	111.05 (14)	H14A—C14—H14B	108.0
			
C7—N4—C1—N1	−179.56 (17)	C9—N6—C7—C6	179.02 (18)
C7—N4—C1—N2	−0.2 (3)	N5—C6—C7—N4	−179.26 (18)
C2—N2—C1—N4	−1.3 (3)	C2—C6—C7—N4	−1.3 (3)
C2—N2—C1—N1	178.04 (17)	N5—C6—C7—N6	−0.4 (2)
C3—N3—C2—N2	−5.9 (3)	C2—C6—C7—N6	177.52 (17)
C3—N3—C2—C6	174.0 (2)	C6—N5—C8—N6	0.1 (2)
C1—N2—C2—N3	−178.59 (17)	C7—N6—C8—N5	−0.4 (2)
C1—N2—C2—C6	1.5 (3)	C9—N6—C8—N5	−178.85 (19)
C2—N3—C3—C4	142.2 (2)	C7—N6—C9—C13	146.99 (18)
C2—N3—C3—C5	72.8 (3)	C8—N6—C9—C13	−34.8 (3)
N3—C3—C4—C5	−107.9 (3)	C7—N6—C9—C10	−95.7 (2)
N3—C3—C5—C4	107.7 (2)	C8—N6—C9—C10	82.6 (2)
C8—N5—C6—C7	0.2 (2)	N6—C9—C10—C11	−110.14 (16)
C8—N5—C6—C2	−177.3 (2)	C13—C9—C10—C11	11.53 (19)
N3—C2—C6—C7	179.77 (18)	C9—C10—C11—C12	−12.79 (19)
N2—C2—C6—C7	−0.4 (3)	C9—C10—C11—C14	105.50 (19)
N3—C2—C6—N5	−2.9 (3)	C14—C11—C12—C13	−110.41 (19)
N2—C2—C6—N5	177.0 (2)	C10—C11—C12—C13	10.0 (2)
C1—N4—C7—N6	−177.05 (18)	C11—C12—C13—C9	−2.7 (2)
C1—N4—C7—C6	1.6 (3)	N6—C9—C13—C12	116.80 (18)
C8—N6—C7—N4	179.31 (18)	C10—C9—C13—C12	−5.9 (2)
C9—N6—C7—N4	−2.1 (3)	C12—C11—C14—O1	179.07 (17)
C8—N6—C7—C6	0.4 (2)	C10—C11—C14—O1	64.7 (2)

### Hydrogen-bond geometry (Å, °)

**Table:**

*D*—H···*A*	*D*—H	H···*A*	*D*···*A*	*D*—H···*A*
N1—H1A···N4^i^	0.88	2.16	3.036 (2)	177
N1—H1B···O1^ii^	0.88	2.36	3.036 (2)	134
N3—H3N···N5^iii^	0.88	2.21	3.016 (2)	152
O1—H1O···N2^iv^	0.84	2.05	2.808 (2)	150

Symmetry codes: (i) −*x*+1, *y*, −*z*+2; (ii) *x*−1/2, *y*−1/2, *z*; (iii) −*x*+1, *y*, −*z*+1; (iv) *x*+1/2, *y*+1/2, *z*.
